# Early wide systolic pressure excursion is associated with clinical outcomes in acute branch atheromatous disease

**DOI:** 10.3389/fneur.2025.1590381

**Published:** 2026-01-02

**Authors:** Zhong Yao, Zhong Fu, LanYing He, YingLin Liu, Jian Wang

**Affiliations:** 1 Department of Neurology, Chengdu Second People's Hospital, Chengdu, Sichuan, China; 2 Department of Neurology, The People’s Hospital of Jianyang, Jianyang, China; 3 Department of Neurology, West China School of Medicine, Sichuan University, Sichuan University Affiliated Chengdu Second People's Hospital, Chengdu, China

**Keywords:** acute ischemic stroke, branch atheromatous disease (BAD), blood pressure excursion, outcome, early neurological deterioration (END)

## Abstract

**Objectives:**

Acute branch atheromatous disease (BAD) is frequently associated with early neurological deterioration (END), which is a significant predictor of poor clinical outcomes. The present study aimed to investigate the relationship between early substantial fluctuations in blood pressure (BP) and both 72-h END and 3-month clinical outcomes in patients diagnosed with acute BAD.

**Methods:**

This investigation included a cohort of 241 patients (mean age 66.10 ± 11.31 years) diagnosed with acute BAD, all of whom underwent comprehensive clinical assessment and continuous blood pressure monitoring. Both maximum and minimum BP levels were systematically recorded during the 72 h following the onset of neurological symptoms. END was defined as an increase of 2 or more points in the National Institutes of Health Stroke Scale (NIHSS) score within 72 h after the onset of stroke.

**Results:**

The results revealed that 23.65% (57/241) of the patients experienced END, while 78.42% (189/241) had favorable clinical outcomes. The patients who experienced END exhibited significantly wider systolic pressure (SP) excursions compared to those without END (*p* = 0.031). In fully adjusted models, after adjusting for potential confounders, no significant association was observed between wide SP excursions and END (*p* = 0.535). Wide SP excursions were associated with a poor prognosis at 3 months in the adjusted models (*p* < 0.05).

**Conclusion:**

Wider SP excursions were independently associated with an elevated risk of poor clinical outcomes in patients with BAD. These findings suggest that excessive fluctuations in early systolic pressure should be controlled during the first 72 h following symptom onset in individuals with acute BAD.

## Introduction

Ischemic stroke is a leading cause of global mortality and disability. Research conducted in real-world environments has highlighted the substantial economic burden of ischemic stroke in China. Over the course of 1 year, the total estimated costs amount to 57,567.48 CNY. This total includes 26,612.67 CNY for direct medical costs, 2,787.56 CNY for direct non-medical expenses, and an additional 28,167.25 CNY for other annual expenditures within the nation (1).

Branch atheromatous disease (BAD), a distinct type of ischemic stroke, is characterized by an intracerebral infarction that affects more than three imaging slices within the territory of basal penetrating arteries or lesions that extend to the base of the pons, as observed through diffusion-weighted magnetic resonance imaging (MRI). This condition occurs in patients who do not have significant arterial stenosis (50% or more) in the supplying vessels of the affected area and do not have atrial fibrillation (2). The primary cause of BAD often involves blockages at the origin of deep brain arteries, which are commonly linked to microatheroma or junctional plaques (3). Clinically, a major concern is that patients with BAD have an increased risk of experiencing early neurological deterioration (END), which tends to worsen more rapidly compared to END in other stroke subtypes (4, 5). Such deterioration can lead to significant patient anxiety. Previous research has indicated that END is not effectively mitigated by traditional stroke interventions such as intravenous thrombolysis and antiplatelet therapy (6–10), frequently resulting in unfavorable functional outcomes (i.e., modified Rankin Scale [mRS] score >1) among individuals with BAD-related strokes.

Approximately 25% of BAD patients experience END and have a poor prognosis (11–13). Although the mechanisms linking BAD and END remain unclear, it is crucial to identify potential risk factors that contribute to END and functional outcomes in this population. One possible factor may be wide excursions in systolic pressure (SP).

Previous studies have established a significant association between wide SP excursions and END following acute ischemic stroke (AIS) (14, 15). For instance, a decrease in systolic pressure of more than 20 mmHg within 3 days has been linked to a lower risk of END in patients with lacunar strokes (15). However, the relationship between fluctuations in maximum and minimum blood pressure (BP) after symptom onset in BAD patients and adverse functional outcomes remains unclear, as this subgroup has not been extensively studied. Consequently, this study aims to investigate the connection between wide systolic pressure excursions, END, and 3-month clinical prognosis in patients diagnosed with BAD.

## Methods

### Study population

This study included patients with AIS who were consecutively admitted to the Second People’s Hospital of Chengdu within 24 h of symptom onset, from May 2012 to December 2017. The diagnosis of AIS was established through diffusion-weighted imaging (DWI) using a 1.5 Tesla Siemens Magnetom Avanto MRI scanner, provided by Siemens Medical Solutions, Erlangen, Germany. Branch atheromatous disease (BAD) involving the lenticulostriate arteries (LSAs) is defined by the presence of infarctions larger than 15 mm in diameter on an axial view, spanning at least three slices, whereas BAD affecting the paramedian pontine arteries (PPAs) is characterized by singular unilateral infarctions that extend to the ventral surface of the pons. The severity of each stroke case was assessed utilizing the National Institutes of Health Stroke Scale (NIHSS) upon hospital arrival. Ethical approval for the study was granted by the hospital’s ethics committee, and written consent was secured from all participants or their designated representatives.

### Inclusion criteria

Patients were included in the study only if they met all of the following criteria: 1. Admission for first-ever AIS within 24 h, with infarctions exceeding 15 mm in diameter on an axial view spanning at least three slices in the lenticulostriate arteries or isolated unilateral infarctions extending to the ventral surface of the pons on DWI. 2. Presence of a single acute ischemic lesion in the cerebral hemisphere corresponding to the clinical presentation. 3. No history of mechanical thrombectomy or administration of thrombolytic agents. All participants received conventional treatment, including aspirin and lipid-lowering drugs, in accordance with the established stroke management protocols.

### Exclusion criteria

The exclusion criteria were as follows: 1. Cardioembolic stroke; 2. cardiac diseases, including acute myocardial infarction, a history of tachyarrhythmia/bradyarrhythmia, or atrial fibrillation; 3. severe pulmonary disease, renal failure(estimated glomerular filtration rate<30 mL/min.1.73 m^2^), or active malignancies; 4. cerebral hemorrhage, fever(≥38 °C), or hypoxia (arterial oxyhemoglobin saturation <90%); and 5. MRI contraindications.

### Neurological assessment and definition of END

Upon admission, an experienced neurologist assessed stroke severity using the NIHSS. For the purpose of our research, END was defined as an increase of 2 or more points in the NIHSS score within 72 h after stroke onset, excluding cases of hemorrhagic conversion or additional infarcts in separate vascular regions. The neurologist conducted NIHSS assessments twice daily during the first 72 h. Following treatment, patients were invited for a personal interview at the 3-month follow-up to assess their outcomes. A favorable prognosis was defined as having an mRS score of 2 or lower at the 3-month check-up.

### Data collection

Upon admission, brain magnetic resonance imaging (MRI) was performed. Baseline BP measurements were obtained by trained nurses. BP was measured with the participant in a supine position using a standard mercury sphygmomanometer and one of four cuff sizes selected according to the participant’s arm circumference. BP measurements were recorded every 2 h for the initial 72-h period. In this study, SP excursion was defined as maximum SP − minimum SP across all valid 2-h measurements during the 72 h after admission.

Demographic data collected included age, sex, body mass index (BMI), diastolic and systolic blood pressures, baseline NIHSS score, and stroke location. In addition, we documented the medical history of tobacco use—defined as either continuous smoking for at least 6 months or daily smoking for a minimum of 6 months—and alcohol consumption—defined as regular intake of alcohol on at least 5 days per week, exceeding 30 grams per day, for a duration of at least 6 months. Within the first 24 h post-admission, a series of laboratory analyses were performed to assess parameters such as white blood cell (WBC) count, neutrophil and lymphocyte counts, monocyte levels, red cell distribution width, mean platelet volume, blood glucose (GLU), glycated hemoglobin (HbA1c), and serum uric acid concentrations.

### Statistical analysis

Initially, the patients were categorized into groups based on the absence or presence of END and prognosis (poor or good). Categorical variables were analyzed using the Pearson *χ*2 test.The student’s *t*-test was used to compare normally distributed variables between two groups, the Mann–Whitney U test was used to compare non-normally distributed variables between two groups. Variables associated with clinical outcomes (END or good prognosis) in the univariate analysis with a *p*-value of < 0.20 were included in the multivariate analysis. We then performed logistic regression analyses to determine the association between risk factors and outcomes (END, good prognosis). The results were expressed as adjusted odds ratios (ORs) with the corresponding 95% confidence interval (CI). The data were analyzed using the SPSS software (SPSS 22.0). *p*-values of <0.05 were considered statistically significant.

## Results

### Characteristics of the study participants

Our study included 241 patients, of whom 35.68% (86) were female, with a mean age of 66.10 ± 11.31 years, ranging from 37 to 88 years. In the study population, 184 patients had a history of hypertension, 82 had a history of diabetes, 76 had a history of hyperlipidemia, 35 were current alcohol drinkers, and 68 were current smokers. A total of 57 (23.65%) patients experienced END.

The baseline characteristics of the patients in the no-END and END groups were compared (Table 1). The END group exhibited a markedly higher proportion of female individuals (OR, 7.51; 95% CI, 0.06–0.009; *p* = 0.006), increased SP (179.18 ± 21.33 vs. 171.54 ± 21.45, *p* = 0.019), and increased DP (104.02 ± 19.17 vs. 100.89 ± 15.24, *p* = 0.024) compared to the no-END group. SP excursions were also higher in the END group (65.70 ± 20.01 vs. 59.29 ± 19.31, *p* = 0.031).

**Table 1:** Comparison of baseline characteristics between patients with no END and END groups.

Characteristics	No END group (184)	END group (57)	OR(95% CI)	p*
Age, y (Mean ± SD)	65.98 ± 11.45	66.46 ± 10.95		0.781
NIHSS score, (Mean ± SD)	3.03 ± 1.94	3.28 ± 1.82		0.382
Maximum SP, (Mean ± SD)	171.54 ± 21.45	179.18 ± 21.33		**0.019**
Minimum SP, (Mean ± SD)	112.36 ± 14.50	113.65 ± 14.46		0.557
Maximum − Minimum SP, (Mean ± SD)	59.29 ± 19.31	65.70 ± 20.01		**0.031**
Maximum DP, (Mean ± SD)	100.89 ± 15.24	104.02 ± 19.17		**0.024**
Minimum DP, (Mean ± SD)	69.80 ± 13.81	69.72 ± 15.21		0.861
Maximum − Minimum DP, (Mean ± SD)	31.08 ± 19.04	34.30 ± 19.49		0.390
Females, *n*(%)	57(30.98)	29(50.88)	7.51(0.06–0.009)	**0.006**
Hypertension, *n*(%)	138(75.00)	46(80.70)	0.78(0.47–0.49)	0.376
Diabetes, *n* (%)	66(35.87)	16(28.07)	1.18(0.33–0.35)	0.278
Hyperlipidemia, *n*(%)	61(33.15)	15(26.32)	0.94(0.41–0.43)	0.332
Current smoking, *n*(%)	53(28.80)	15(26.32)	0.13(0.73–0.75)	0.715
Current alcohol drinking, *n*(%)	30(16.30)	5(8.77)	1.99(0.19–0.21)	0.158
Dual antiplatelet+argatroban, *n*(%)	24(13.04)	3(5.26)	2.65(0.11–1.28)	0.104
GLU, mmol/L (Mean ± SD)	7.20 ± 3.82	7.10 ± 3.57		0.864
TG, mmol/L (Mean ± SD)	1.93 ± 2.01	1.61 ± 0.86		0.244
Tcho, mmol/L (Mean ± SD)	4.87 ± 1.36	4.83 ± 1.51		0.834
HDL-C, mmol/L (Mean ± SD)	1.20 ± 0.56	1.18 ± 0.34		0.871
LDL-C, mmol/L (Mean ± SD)	2.90 ± 0.97	3.07 ± 1.19		0.254
BUN, mmol/L (Mean ± SD)	5.57 ± 1.65	5.19 ± 1.47		0.120
HbAlc, % (Mean ± SD)	6.97 ± 2.07	6.75 ± 1.78		0.461
Length of stay, d (Mean ± SD)	12.08 ± 7.88	12.71 ± 2.65		0.557
Location of stroke, *n*(%)				
Basal ganglia, *n*(%)	105(57.07)	31(54.39)	0.90(0.49–1.63)	0.772
Pons, *n*(%)	79(42.93)	26(45.61)	0.90(0.49–1.63)	0.772
Pre-stroke medications use				
Antiplatelet, *n*(%)	4(2.17)	1(1.75)	0.04(1.00–1.00)	0.846
Tatin, *n*(%)	4(2.17)	1(1.75)	0.04(1.000–1.000)	0.846
3 months mRS ≤ 2, *n*(%)	167(90.76)	22(38.60)	69.98(0.000–0.000)	**<0.001**

*Comparison between no END and END groups. Continuous variables are expressed as mean ± standard deviation (SD). Categorical variables are expressed as frequency (%) for *p* values, Pearson χ^2^ test, Fisher exact 2-sided test, and Student *t* test were used when appropriate. Distributions of continuous variables were determined by the Kolmogorov–Smirnov test, Mann–Whitney two sample test was applied in case of non-normal distributions. Bold indicates *p*-values less than 0.05.

### Multivariable models of the association between risk factors and END

In unadjusted models, there was an association between female sex and END (OR, 2.31; 95% CI, 1.26–4.23, *p* = 0.007). Variables associated with END in the univariate analysis with a *p*-value of < 0.20 were included in the multivariate analysis. In the multivariable model adjusted for maximum SP, maximum DP, SP excursion (maximum–minimum SP), sex, current alcohol use, dual antiplatelet+argatroban, and BUN, female sex remained a significant predictor of END (adjusted OR, 2.23; 95% CI, 1.16–4.30; *p* = 0.018) (Table 2).

**Table 2:** Multivariable models showing the association between risk factors and END.

Group	OR (95% CI)	p*
Maximum SP	1.01 (0.99–1.04)	0.418
Maximum DP	1.01(0.98–1.03)	0.481
Maximum − Minimum SP	1.01(0.98–1.03)	0.527
Females	2.23(1.16–4.30)	**0.018**
Current alcohol drink	0.75(0.26–2.18)	0.527
Dual antiplatelet+argatroban	0.35(0.10–1.24)	0.104
Blood urea nitrogen (BUN)	0.81(0.66–1.01)	0.066

*Multivariable adjusted for maximum SP, maximum DP, maximum − minimum SP, gender, current alcohol drink, dual antiplatelet + argatroban, BUN. Bold indicates *p*-values less than 0.05.

### Univariable models for predictors of good prognosis

A total of 189(189/241, 78.42%) patients had a good prognosis at 3 months (Table 3). At baseline, the patients with a good prognosis had a significantly lower proportion of female individuals (31.22% vs. 51.92%, *p* = 0.06), lower pre-stroke antiplatelet use (5.77% vs. 1.06%, *p* = 0.035), lower pre-stroke statin use (5.77% vs. 1.06%, *p* = 0.035), lower NIHSS score (2.63 ± 1.52 vs. 4.73 ± 2.26, *p* < 0.001), lower maximum SP (170.10 ± 20.99 vs. 185.13 ± 19.87, *p* < 0.001), lower SP excursion (57.43 ± 18.77 vs. 73.08 ± 17.86, *p* < 0.001), and lower GLU (6.85 ± 3.19 vs. 8.35 ± 5.21, *p* = 0.010).

**Table 3:** Comparison of baseline characteristics between patients with poor prognosis and good prognosis groups.

Variables	Poor prognosis group (52)	Good prognosis group (189)	OR(95% CI)	p*
Age, y(Mean ± SD)	66.42 ± 10.53	66.00 ± 11.54		0.812
NIHSS score, (Mean ± SD)	4.73 ± 2.26	2.63 ± 1.52		<0.001
Maximum SP, (Mean ± SD)	185.13 ± 19.87	170.10 ± 20.99		<0.001
Minimum SP, (Mean ± SD)	112.06 ± 15.19	112.83 ± 14.30		0.734
Maximum − Minimum SP, (Mean ± SD)	73.08 ± 17.86	57.43 ± 18.77		<0.001
Maximum DP, (Mean ± SD)	104.56 ± 16.32	100.82 ± 16.20		**0.086**
Minimum DP, (Mean ± SD)	68.81 ± 14.36	70.05 ± 14.08		0.958
Maximum − Minimum DP, (Mean ± SD)	35.75 ± 20.13	30.77 ± 18.80		**0.095**
Females, *n*(%)	27(51.92)	59(31.22)	0.42(0.23–0.79)	**0.006**
Hypertension, *n*(%)	42(80.77)	142(75.13)	0.72(0.34–1.55)	0.397
Diabetes, *n*(%)	17(32.69)	65(34.39)	1.8(0.56–2.07)	0.819
Hyperlipidemia, *n*(%)	13(25.00)	63(33.33)	1.50(0.75–03.01)	0.252
Current smoking, *n*(%)	10(19.23)	58(30.68)	1.86(0.87–3.96)	0.104
Current alcohol drinking, *n*(%)	5(9.62)	30(15.87)	1.77(0.65–4.83)	0.257
Antiplatelet+argatroba, *n*(%)	25(48.08)	3(1.59)	3.81(0.87–16.65)	0.058
GLU, mmol/L (Mean ± SD)	8.35 ± 5.21	6.85 ± 3.19		**0.010**
TG, mmol/L (Mean ± SD)	1.94 ± 2.42	1.83 ± 1.61		0.694
Tcho, mmol/L (Mean ± SD)	5.08 ± 1.77	4.80 ± 1.27		0.207
HDL-C, mmol/L (Mean ± SD)	1.15 ± 0.28	1.20 ± 0.56		0.491
LDL-C, mmol/L (Mean ± SD)	3.05 ± 1.15	2.91 ± 0.99		0.394
BUN, mmol/L (Mean ± SD)	5.61 ± 1.78	5.44 ± 1.57		0.522
HbAlc(%), (Mean ± SD)	7.27 ± 2.30	6.82 ± 1.90		0.156
Location of stroke, *n*(%)				
Basal ganglia, *n*(%)	32(61.54)	104(55.03)	0.77(0.41–1.43)	0.402
Pons, *n*(%)	20(38.46)	85(45.95)	0.77(0.41–1.43)	0.402
Length of stay, d(Mean ± SD)	12.08 ± 7.88	12.71 ± 2.65		0.557
END, *n*(%)	35(67.31)	22(42.31)	0.064(0.031–0.133)	<0.001
Pre-stroke medications use				
Antiplatelet, *n*(%)	3(5.77)	2(1.06)	0.18(0.03–1.08)	**0.035**
Tatin, *n*(%)	3(5.77)	2(1.06)	0.18(0.03–1.08)	**0.035**

*Comparison between poor prognosis and good prognosis groups. Continuous variables are expressed as mean ± standard deviation (SD). Categorical variables are expressed as frequency (%) for *p* values, Pearson χ^2^ test, Fisher exact 2-sided test, and Student *t* test were used when appropriate. Distributions of continuous variables were determined by the Kolmogorov–Smirnov test, Mann–Whitney two sample test was applied in case of non-normal distributions. Bold indicates *p*-values less than 0.05.

### Multivariable models of the association between factors and good prognosis

In unadjusted models, there was a negative association between SP excursion and good prognosis (aOR, 0.76; 95% CI, 0.55–0.97, *p* < 0.001). In the multivariable logistic regression model, after adjustment for maximum SP, maximum DP, SP excursion, DP excursion, sex, current alcohol use, dual antiplatelet+argatroban, NIHSS score, GLU, HbAlc on admission, and pre-stroke statin and antiplatelet use (Model 1), SP excursion (aOR, 0.77; 95% CI, 0.54–0.99; *p* = 0.015), female sex (aOR, 0.34; 95% CI, 0.14–0.75; *p* = 0.021), and lower NIHSS scores on admission(aOR, 1.42; 95% CI, 1.06–2.34; *p* < 0.001) were significant predictors of good prognosis at 3 months. Even when END was included in the multivariate logistic regression (Model 2), after adjustment for SP excursion, sex, current alcohol use, dual antiplatelet+argatroban, NIHSS score, GLU, HbAlc on admission, and pre-stroke antiplatelet and statin use, the results showed that SP excursion (aOR, 0.86; 95% CI, 0.73–0.97; *p* = 0.037), female sex (aOR, 0.54; 95% CI, 0.34–0.75; *p* = 0.041), lower NIHSS scores on admission(aOR, 1.36; 95% CI, 1.04–2.12; *p* < 0.001), and END (aOR, 0.074; 95% CI, 0.03–0.14; *p* < 0.001) were significant predictors of good prognosis at 3 months (Table 4).

**Table 4:** Multivariable models showing the association between risk factors and good prognosis.

Variables	OR (95% CI)	p*
Model 1		
Maximum SP	0.99(0.96–1.02)	0.558
Maximum DP	1.01(0.98–1.05)	0.413
Maximum − Minimum SP	0.77(0.54–0.99)	**0.015**
Maximum − Minimum DP	0.99(0.96–1.01)	0.309
Females	0.34(0.14–0.75)	**0.021**
Current alcohol drink	1.44(0.34–6.06)	0.616
Dual antiplatelet+argatroban	2.72(0.53–14.05)	0.233
Lower NIHSS on admission	1.42(1.06–2.34)	<0.001
GLU on admission	0.89(0.77–1.03)	0.111
HbAlc on admission	1.09(0.83–1.44)	0.537
Pre-stroke antiplatelet	0.76(0.08–7.20)	0.809
Pre-stroke tatins	0.76(0.08–7.20)	0.809
Model 2		
Maximum − Minimum SP	0.86(0.73–0.97)	**0.037**
Females	0.54(0.34–0.75)	**0.041**
Current alcohol drink	1.03(0.19–5.62)	0.972
Dual antiplatelet+argatroban	201(0.30–13.59)	0.474
Lower NIHSS on admission	1.36(1.04–2.12)	<0.001
GLU on admission	0.90(0.75–1.07)	0.239
HbAlc on admission	1.07(0.75–1.53)	0.537
END	0.074(0.03–0.14)	<0.001
Pre-stroke antiplatelet	0.21(0.02–2.70)	0.229
Pre-stroke tatins	0.21(0.02–2.70)	0.229

*Multivariable adjusted for maximum SP, maximum DP, maximum − minimum SP, maximum − minimum DP, gender, current alcohol drink, dual antiplatelet+argatroban, NIHSS, GLU, HbAlc on admission, END, pre-stroke tatins and antiplatelet use. Bold indicates *p*-values less than 0.05.

## Discussion

Our findings indicate that female sex is associated with END and that greater SP variability, higher NIHSS scores on admission, and female sex are associated with an unfavorable prognosis at 3 months in patients with BAD.

To the best of our knowledge, the present study is one of the few studies to explore the risk factors of END in BAD. Although several studies have examined clinical outcomes and risk factors in male and female individuals with ischemic stroke, their findings have been inconsistent (16–19). Most studies have reported no significant difference in recurrence risk between male and female patients (6, 11, 12, 20). A substantial body of research indicates that advancing age is linked to a heightened state of persistent, low-level inflammation, termed “inflammaging,” which predisposes older adults to adverse health effects following ischemic stroke. Post-stroke, female individuals tend to experience greater disability and a lower quality of life compared to their male counterparts in the same age group (20). Before menopause, female individuals appear to have some degree of protection against stroke, while male individuals exhibit higher stroke incidence and poorer rehabilitation outcomes from childhood through early adulthood. This disparity narrows and eventually reverses in middle age, likely due to the cessation of menstruation in female individuals and the subsequent decline in sex hormones, particularly estrogens (21). In this study, we found that female sex was an independent risk factor for END and poor prognosis in BAD. We speculate that this may be related to the reduced protective effects of hormones and the increased vascular inflammatory response in female individuals during the perimenopausal period.

Following a stroke, an immediate increase in blood pressure might partially offset the effects of ischemia by enhancing blood flow through collateral vessels. However, this is accompanied by unavoidable blood pressure variations that could potentially interfere with the consistent perfusion needed in the ischemic region (22, 23). Consequently, blood pressure adjustments after a stroke are inherently flawed compensatory mechanisms that may, at times, worsen the condition of the vulnerable ischemic brain (24, 25). In our study, the results showed that there was no association between SP fluctuations and END. However, at baseline, SP fluctuations were greater in the END group compared to the no-END group, which may be related to the small sample size.

Patients with poor prognosis at 3 months after AIS had wider BP excursion within 72 hours of admission. The results of this study were inconsistent with previous studies (25–28), the reason for this difference may be related to the inconsistent time limits of blood pressure fluctuations and subtypes of AIS. The patients included in our study had BAD, while most other studies focused on lacunar cerebral infarction, especially cases involving lenticulostriate artery occlusion. Stroke in these two types of patients may have different pathogenesis.

Recent studies have reported that the pathology of BAD is related to atherosclerosis and its progression (29). The adverse prognosis associated with fluctuating systolic blood pressure may result from impaired vascular reactivity, thickening of the arterial hyaline wall, and degeneration of smooth muscle, making brain tissue more dependent on systemic blood pressure for perfusion. If blood pressure drops suddenly, patients are more susceptible to hypoperfusion in the ischemic area, causing the occurrence of END and leading to a poor prognosis (29).

This study found that the patients in the END group exhibited higher blood pressure fluctuations, but the effect disappeared after adjusting for confounders, which may be related to the small sample size. END is a well-established risk factor for poor prognosis at 3 months following AIS, as demonstrated in numerous studies (30, 31). The current study revealed that, in the patients with BAD, even after adjusting for END, wide systolic pressure excursions remained an independent risk factor for poor prognosis at 3 months. This effect may be related to impaired vascular self-regulation.

## Conclusion

In conclusion, our study revealed a significant positive correlation between systolic blood pressure excursions and clinical outcomes, with female individuals being more prone to END and poor prognosis in BAD. Our findings confirm the importance of blood pressure stabilization in the early stages of BAD, given the increased risk of a poor prognosis after stroke. Early monitoring of blood pressure may have potential predictive value for risk stratification in patients with BAD. Patients admitted to the stroke unit should undergo routine ambulatory blood pressure monitoring and be closely observed for blood pressure fluctuations. Our results may help guide the management of patients with BAD.

### Limitations

There are several limitations of this study that should be noted. First, based on DWI data for BAD, we did not classify the responsible vascular lesions using high-resolution magnetic resonance vessel wall imaging. Second, the study did not explore the optimal blood pressure management strategies for patients with BAD. Third, there was no analysis of the categories of drugs used for blood pressure control in acute stroke. Follow-up DWI data after exacerbation were not obtained. As this was a single-center study, the generalizability of our findings requires further research. In the future, we plan to conduct multi-center studies to determine the optimal blood pressure management strategies following BAD. Despite these limitations, the findings of this study provide valuable guidance for the management of blood pressure in patients with BAD.

## Data Availability

The original contributions presented in the study are included in the article/supplementary material, further inquiries can be directed to the corresponding authors.
